# The prediction role of perfectionism on exercise addiction in college students: chain-mediated effects of self-esteem and social anxiety

**DOI:** 10.3389/fpsyg.2026.1751643

**Published:** 2026-02-12

**Authors:** Jun Gao, Tong Liu, Shuting Huang, Zhonggen Yin

**Affiliations:** 1 Department of Physical Education, Southwest Jiaotong University Hope College, Chengdu, Sichuan, China; 2 School of Sports Training, Chengdu Sport University, Chengdu, Sichuan, China; 3 Chongqing Yucai Secondary School, Jiulongpo, Chongqing, China; 4 College of Physical Education and Health Management, Chongqing University of Education, Nanan, Chongqing, China

**Keywords:** college student, exercise addiction, perfectionism, self-esteem, social anxiety

## Abstract

**Objective:**

This study aimed to test a chain mediation model examining whether self-esteem and social anxiety chain mediate the relationship between perfectionism and exercise addiction risk among Chinese college students.

**Methods:**

Using a cross-sectional design, 1,823 college students (45.75% male, 54.25% female) from 15 provinces in China were recruited via convenience sampling. Standardized scales were used to measure perfectionism, self-esteem, social anxiety, and the risk of exercise addiction. Statistical methods, including the Bootstrap approach, were employed to examine the mediating effects and analyze the relationships among variables.

**Results:**

Perfectionism was positively associated with exercise addiction risk (*β* = 0.56, *p* < 0.001). It was also linked to exercise addiction through three indirect paths: (1) the independent mediating path via self-esteem (*β* = 0.03, *p* < 0.001); (2) the independent mediating path via social anxiety (*β* = 0.02, *p* < 0.001); and (3) the chain mediating path via self-esteem and then social anxiety (*β* = 0.01, *p* < 0.001). The total indirect effect was 0.06, accounting for 42.86% of the total effect.

**Conclusion:**

This study suggests that among college students, perfectionism is not only directly associated with a higher risk of exercise addiction but may also increase risk indirectly through the independent and sequential roles of low self-esteem and high social anxiety. These findings provide a preliminary basis for understanding the psychological mechanisms of exercise addiction. Future longitudinal studies are needed to further clarify the temporal and causal relationships among these variables.

## 1 Introduction

The role of physical activity in enhancing health is well-established. However, excessive and compulsive exercise, often termed “exercise addiction,” can lead to significant physical and psychological harm, including injury, social impairment, and distress (Weinstein and Szabo, 2023). It should be noted that ‘exercise addiction’ is not a formal diagnosis in the Diagnostic and Statistical Manual of Mental Disorders (APA, 2013), but is used here as a well-established construct in behavioral addiction research to describe a pattern characterized by core components such as salience, tolerance, and withdrawal. In this study, we measure this construct as a continuous indicator of exercise addiction risk. College students are considered a high-risk group due to academic pressures and societal focus on body image (Li et al., 2015).

Perfectionism, characterized by excessively high personal standards and self-criticism, has been consistently identified as a key antecedent to exercise addiction (Caponnetto et al., 2021; Çetin et al., 2021). Theoretical models suggest that this relationship is not direct but operates through intermediary psychological mechanisms. Specifically, perfectionism may erode self-esteem (Bardone-Cone et al., 2007), which in turn may heighten social anxiety, particularly in contexts where social evaluation is salient (Clark and Wells, 1995).

However, critical gaps remain. First, while perfectionism, self-esteem, and exercise addiction are established correlates, the theoretical rationale and empirical evidence for including general social anxiety (beyond social physique anxiety) in this chain remains underdeveloped. Second, most prior research has examined these variables in isolation or tested simple mediation models, neglecting the possibility of a sequential chained mediation process (perfectionism → self-esteem → social anxiety → exercise addiction). Furthermore, this integrated mechanism has not been sufficiently examined in non-clinical populations, particularly within specific cultural contexts such as China, where collectivist values and intense pressures related to academics and physical appearance may uniquely amplify these psychological pathways.

To address these gaps, this cross-sectional study tests the proposed chained mediation model in a sample of Chinese college students. Its contributions are threefold. First, it provides an initial empirical test of the chain mediation pathway (perfectionism → self-esteem → social anxiety → exercise addiction), moving beyond isolated associations. Second, it integrates self-regulation theory to offer a novel mechanistic explanation, positing that social anxiety depletes the cognitive resources necessary for behavioral control. Third, it examines this model within a specific cultural context (non-clinical Chinese students), generating insights that may differ from Western-centric findings. These cross-sectional findings contribute a preliminary understanding of the psychological architecture of exercise addiction.

## 2 Literature review and hypothesis development

### 2.1 Perfectionism and exercise addiction

The following review summarizes literature on perfectionism and exercise addiction (often studied as a continuum of risk in non-clinical populations). Perfectionism is defined as a personality trait characterized by setting excessively high standards for oneself, coupled with harsh self-criticism (Frost et al., 1990). It encompasses two dimensions: self-oriented perfectionism (demanding excessively high personal standards) and socially prescribed perfectionism (perceiving high expectations from others). Empirical evidence suggests that individuals with high perfectionism tend to view exercise as a tool for weight or body shape control, thereby exacerbating compulsive exercise behaviors (Caponnetto et al., 2021). For instance, a systematic review by Caponnetto highlighted a significant association between perfectionism and exercise addiction, particularly when individuals link exercise performance to self-worth (e.g., believing that “only thinness is recognized”) (Caponnetto et al., 2021). Lichtenstein further identified that perfectionists’ “maladaptive pursuit of excellence” may lead to a loss of control over exercise, persisting even when facing physical injuries or social conflicts (Lichtenstein et al., 2017). Specifically, perfectionists’ “fear of failure” and “achievement-oriented mindset” may drive excessive exercise to alleviate anxiety or maintain self-worth (Meyer et al., 2011). For example, among athletes, perfectionism is significantly associated with exercise addiction (McNamara and McCabe, 2012). Individuals with high perfectionism are more likely to use exercise to compensate for self-perceived deficits (Çetin et al., 2021). Similarly, Davis found that female college students’ perfectionism scores were positively correlated with exercise addiction, and this relationship was moderated by exercise habits (Davis, 1990). In contrast, Hagan demonstrated that college students with high exercise addiction scores exhibited significantly higher perfectionism levels than those with low scores (Hausenblas and Giacobbi, 2004). Collectively, these findings suggest that perfectionism serves as a critical antecedent to exercise addiction. Therefore, we propose Hypothesis H1: Perfectionism positively predicts exercise addiction.

### 2.2 Perfectionism, self-esteem, and exercise addiction

Self-esteem encompasses an individual’s global evaluation of self-worth (i.e., self-acceptance independent of achievements or appearance) and specific self-evaluations (Dutton and Brown, 1997). Empirical studies consistently link low self-esteem to exercise addiction (Chen, 2016; Cosh et al., 2023; Corazza et al., 2019). For example, a cross-sectional study among female university students revealed a significant negative correlation between exercise addiction and self-esteem (Olave et al., 2025). Self-esteem also mediates the relationship between perfectionism and behavioral addictions (Ertl et al., 2018). Specifically, perfectionists often experience diminished self-esteem due to discrepancies between their actual performance and unattainable ideals (Lowery et al., 2005). Bardone-Cone demonstrated that perfectionists’ failure to meet self-imposed high standards triggers negative self-appraisals (e.g., low self-esteem) (Bardone-Cone et al., 2007), which may indirectly drive maladaptive behaviors such as eating disorders or compulsive exercise.

Individuals with low self-esteem are more likely to adopt avoidant coping strategies (e.g., diverting attention from stressors through excessive exercise) to manage negative emotions (Lazarus, 1966). Building on this general tendency, we posit that within the framework of our model, the negative emotions particularly relevant are those arising from perceived social evaluation, that is, social anxiety. A meta-analysis further confirmed that higher perfectionism correlates with lower self-esteem (Khossousi et al., 2024). However, the association between self-esteem and exercise addiction may vary depending on self-esteem type (e.g., stable vs. fragile). Notably, recent research identifies a dual-risk mechanism: while high self-esteem is generally protective, individuals who overly rely on exercise to maintain self-worth (e.g., believing “exercise makes me valuable”) may ignore the physical and psychological harms of over-exercising (Ertl et al., 2018). This non-linear mediation pathway highlights the need to contextualize findings within cultural frameworks that prioritize body image (e.g., East Asian societies). It is important to note that the relationship between perfectionism and self-esteem is theoretically complex and potentially bidirectional. While the present study focuses on perfectionism as an antecedent to self-esteem—consistent with the theoretical view that chronic failure to meet unrealistic standards erodes one’s sense of self-worth (Khossousi et al., 2024; Piotrowski et al., 2023)—an alternative perspective suggests that low self-esteem may also predispose individuals to develop perfectionistic tendencies as a compensatory strategy to gain validation and worth (Stricker and Preckel, 2022; Shahyad et al., 2017). However, within the specific context of exercise addiction and for the purposes of testing our proposed mediation model, we position perfectionism as the predisposing personality trait that influences subsequent levels of self-esteem. Our model aims to test this specific cognitive-affective pathway. Therefore, we propose Hypothesis H2: Self-esteem mediates the relationship between perfectionism and exercise addiction risk.

### 2.3 Perfectionism, social anxiety and exercise addiction

Beyond the mediating role of self-esteem, social anxiety may constitute a critical mechanism linking perfectionism to exercise addiction. Social anxiety, defined as a persistent fear of negative evaluation in social contexts (Clark and Wells, 1995), is prevalent among college students.

The inclusion of social anxiety in our model is supported by self-regulation theory (Carver and Scheier, 1998; Baumeister et al., 2007). This theory posits that conscious self-control depends on finite mental resources. The state of social anxiety, characterized by persistent worry and preoccupation with potential social threats, consumes substantial self-regulatory resources as individuals attempt to manage their anxious thoughts and feelings (Spurr and Stopa, 2002). Consequently, when these resources are depleted, the capacity for conscious behavioral monitoring and moderation becomes compromised (Boekaerts et al., 2000).

Within our proposed sequence, perfectionism and diminished self-esteem establish a foundation of heightened self-monitoring and emotional vulnerability. Social anxiety then critically amplifies this self-regulatory depletion. Within the Chinese cultural context, where social harmony and adherence to collective standards (including physical appearance norms) are highly valued, the fear of negative social evaluation may be particularly salient. This cultural characteristic potentially strengthens the pathway from social anxiety to exercise addiction as a compensatory strategy. Under conditions of impaired self-control, exercise, which provides immediate, predictable feedback and potent mood-altering effects, may readily shift from a regulated activity to a maladaptive and compulsive coping strategy aimed at alleviating the distress of social anxiety. This explanatory mechanism aligns with established research linking self-regulatory failure due to social anxiety to other compulsive behaviors, for example, problematic buying and emotional eating (Harnish et al., 2019; Wang et al., 2024). Therefore, we propose Hypothesis H3: Social anxiety mediates the relationship between perfectionism and exercise addiction risk.

### 2.4 Perfectionism, self-esteem, social anxiety, and exercise addiction

Although the studies have independently established links between perfectionism, self-esteem, social anxiety, and exercise addiction, a critical gap remains in understanding how these psychological constructs interact in concert to drive addictive exercise behaviors. Most prior research has examined these variables in isolation or through simple mediation models (Costa et al., 2016; Biggs et al., 2024; Mavrandrea and Gonidakis, 2023), potentially overlooking the more complex, cascading psychological processes that underlie the development of exercise addiction. Specifically, it remains unclear whether self-esteem and social anxiety operate as chain mediators in the pathway from perfectionism to exercise addiction. Testing this chained mediation model is crucial because it can reveal the multifaceted psychological mechanism—whereby perfectionistic concerns sequentially diminish self-esteem, which in turn exacerbates social anxiety, ultimately leading to exercise as a maladaptive coping strategy. This integrated approach moves beyond examining isolated pathways and provides a more holistic theoretical framework for understanding the etiology of exercise addiction. Therefore, the primary innovation of this study lies in its integrative examination of this hypothesized chain of psychological events. Building on prior evidence that self-esteem and social anxiety independently mediate the relationship between perfectionism and exercise addiction, we further propose a chained mediation mechanism. Empirical studies consistently demonstrate a robust negative correlation between self-esteem and social anxiety across diverse populations. For example, a cross-sectional survey of Chinese university students revealed that lower self-esteem significantly predicted higher social anxiety levels (Tan et al., 2016). Similarly, longitudinal data indicated that college students with low self-esteem were more likely to develop social fears (Lv et al., 2024). This pattern extends to non-Western contexts, as evidenced by Ayed, who identified a negative correlation between self-esteem and social anxiety among Palestinian nursing undergraduates (Ayed et al., 2024). Collectively, these findings suggest that perfectionism may drive exercise addiction through the synergistic effects of diminished self-esteem and heightened social anxiety.

This study hypothesizes that self-esteem and social anxiety chain mediate the perfectionism-exercise addiction link. Specifically, social anxiety not only directly mediates this relationship but also functions within a cognitive-emotional-behavioral cascade initiated by low self-esteem. Perfectionists, constrained by their inability to meet self-imposed standards, experience chronic self-devaluation. Individuals with low self-esteem tend to catastrophize the risk of negative social evaluations, thereby amplifying anxiety and perpetuating compulsive exercise as a maladaptive coping strategy (Ashby and Rice, 2002). Consequently, we propose Hypothesis H4: Self-esteem and social anxiety form a chained mediation pathway between perfectionism and exercise addiction.

**Figure 1:**
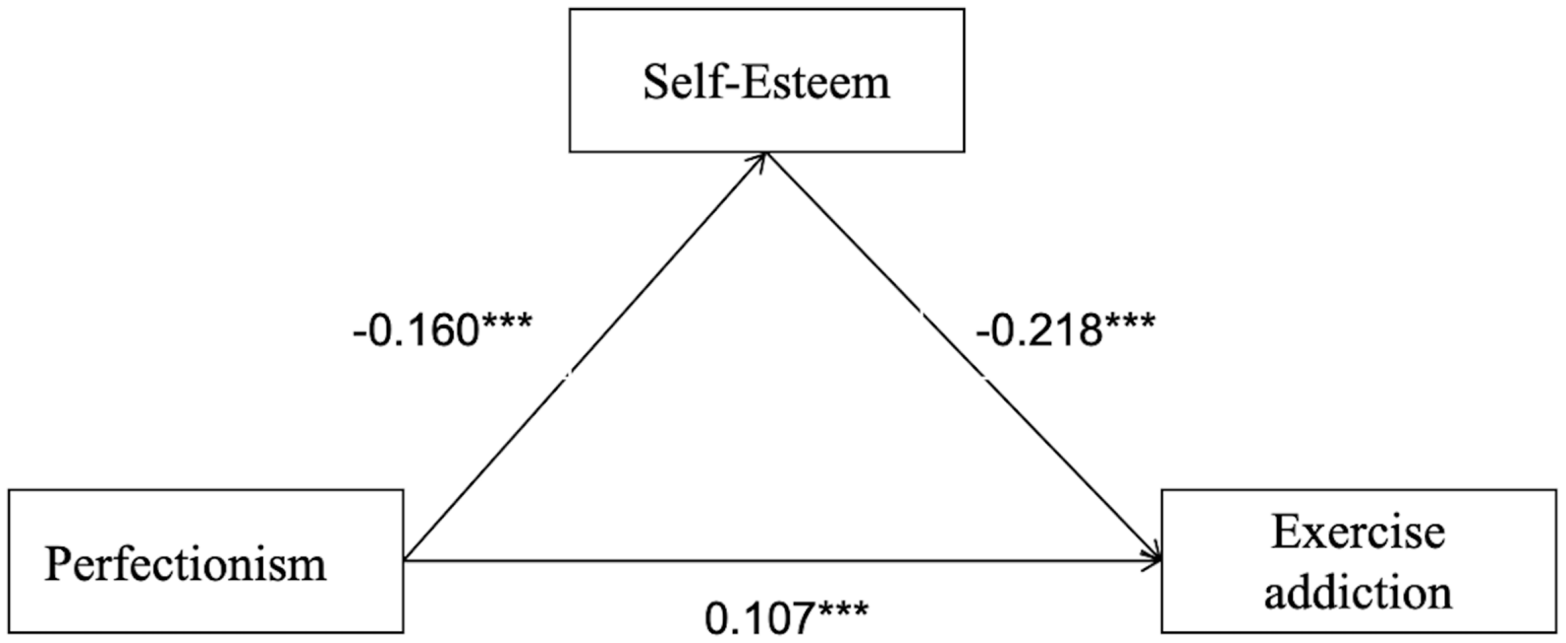
The chain mediation effect model. The symbol *** indicates statistical significance at *p* < 0.001.

Perfectionism → self-esteem → social anxiety → exercise addiction (Figure 1).

## 3 Methodology

### 3.1 Procedures and participants

This study used a cross-sectional design to recruit a sample of 1,900 adult college students from 15 provinces across five regions of China (eastern, central, western, southern, and northern) between March 1–31, 2025, using a convenience sampling method. To implement this, our research team first contacted PE department heads in these universities, explaining the study’s purpose and procedure. Upon their agreement, we enlisted the help of PE teachers. Prior to distribution, these teachers received standardized training from our research team on the administration protocol, which emphasized the voluntary nature of participation, the right to withdraw, and the importance of maintaining confidentiality. The teachers then distributed the electronic questionnaire link to their students during class or through university online student groups. It is important to acknowledge the limitations of this approach. Convenience sampling limits the generalizability of findings beyond similar university contexts. The cross-sectional nature of the data precludes causal inference regarding the proposed mediation pathways. Furthermore, reliance on self-report measures may introduce common method variance, although steps were taken to mitigate this risk (see Section 3.4). The use of convenience sampling and the measure of exercise addiction risk warrant explanation. Convenience sampling was chosen for pragmatic reasons (e.g., accessibility of the population, resource constraints) and is appropriate for initial model testing in behavioral research, where the goal is to examine relationships between variables rather than establish prevalence. To enhance diversity, sampling occurred across 15 provinces. Exercise addiction was operationalized as a continuous measure of risk (via the EAI) in line with behavioral addiction research, not as a clinical diagnosis.

A prior sample size calculation was performed using G*Power 3.1. Based on a linear multiple regression analysis with a significance level (*α*) of 0.05, a statistical power of 0.80, three predictor variables, and a small effect size (*f*^2^ = 0.02), the minimum required sample size was 959. To account for potential invalid questionnaires and participant attrition, we targeted a sample size of 1,200. Data were collected via an electronic questionnaire administered through an online platform. The questionnaire comprised five sections. Basic demographics: Grade, gender, height, weight; Exercise Addiction Inventory (EAI); Chinese Frost Multidimensional Perfectionism Scale (CFMPS); Social Anxiety Scale (SAS); Rosenberg Self-Esteem Scale (RSES). Inclusion criteria for participation were: (1) being a full-time undergraduate student enrolled in one of the sampled universities, and (2) providing informed electronic consent to participate in the study. Exclusion criteria were: (1) severe physical illness, (2) history of psychiatric diagnosis, or (3) recent psychotherapy (within 3 months). All participants provided electronic informed consent prior to data collection (see Section 3.2 for details). Following data collection, 77 invalid responses were excluded based on predefined criteria: (1) abnormally short or long completion times (<60 s or >3,600 s), (2) non-engaged response patterns (e.g., repetitive or inconsistent answers), and (3) self-report of meeting any of the exclusion criteria. The final sample consisted of 1,823 participants (effective response rate: 95.95%). The sample had a mean age of 20.5 years (SD = 1.27; range: 18–24) and a mean BMI of 21.104 (SD = 3.25; range: 14.17–38.06). Demographically, the sample included 989 (54.25%) females and 834 (45.75%) males. By academic standing, there were 715 (39.22%) freshmen, 450 (24.68%) sophomores, 381 (20.90%) juniors, and 277 (15.19%) seniors. Regarding household registration, 1,141 (62.59%) participants were from rural areas, and 682 (37.41%) were from urban areas. We note that other potentially relevant variables, such as socioeconomic status, specific personality traits (e.g., neuroticism), and social media usage, were not assessed. Their omission is a limitation, as they could act as confounding factors. After data collection, the data were anonymized to ensure that participants could not be personally identified. Following data collection, the anonymized dataset will be securely archived and made available for research purposes for a minimum of 5 years post-publication, in compliance with institutional ethical guidelines and data management policies. Access to the data will be granted upon reasonable request to qualified researchers, subject to a formal data sharing agreement that ensures participant confidentiality and adherence to the original informed consent terms.

### 3.2 Ethics approval and consent to participate

This study was conducted in accordance with the ethical standards outlined in the Declaration of Helsinki. Ethical approval was obtained from the Ethics Committee of Chongqing University of Education (Approval No. CQUE-LL20250301002). Prior to participation, all participants provided electronic informed consent through an online platform. The consent form explicitly explained the study’s purpose, voluntary nature of participation, confidentiality protocols, and the right to withdraw at any stage without penalty. Data were anonymized during collection and analysis to ensure participant confidentiality. No personally identifiable information was retained in the final dataset.

### 3.3 Measurement tools

#### 3.3.1 Perfectionism scale

Perfectionism was assessed using the Chinese Frost Multidimensional Perfectionism Scale (Frost et al., 1990; Chinese version: Fei, 2006). This 27-item instrument is a widely used and validated adaptation in the Chinese context, demonstrating a similar factor structure and satisfactory reliability (subscale *α* = 0.64–0.81) in Chinese populations (Fei, 2006). It measures multiple dimensions, including Concern Over Mistakes (e.g., “If I fail at work/school, I am a failure as a person”), Personal Standards (e.g., “I set higher goals than most people”), Parental Expectations, and Doubts About Actions. Responses are recorded on a 5-point Likert scale ranging from 1 (“not at all”) to 5 (“completely”), yielding a total score range of 27 to 135, with higher scores indicating higher levels of perfectionism. The original FMPS developed by Frost et al. demonstrated excellent psychometric properties. The internal consistency for the subscales ranged from 0.77 to 0.93, and the total scale *α* was 0.90. Construct validity was established through significant correlations with measures of psychopathology, compulsivity, and procrastination, confirming its utility in assessing multidimensional perfectionism (Frost et al., 1990). The Chinese version used in this study has also been validated, showing a similar factor structure and satisfactory reliability (subscale *α* = 0.64–0.81) in Chinese populations (Fei, 2006). Following validation, this scale demonstrated good reliability and validity in the present study (Cronbach’s *α* = 0.95; KMO = 0.97, cumulative explained variance 62.56%).

#### 3.3.2 Self-esteem scale

The Rosenberg Self-Esteem Scale (RSES) was employed to measure global self-worth and self-acceptance (Rosenberg, 1989; Wang et al., 1999). The scale contains 10 items (e.g., “Overall, I am satisfied with myself”), with 5 reverse-scored items (items 3, 5, 8, 9, and 10). Two other typical items include: “I feel that I have a number of good qualities” (positively worded) and “I certainly feel useless at times” (reverse-scored). Responses were recorded on a 4-point Likert scale (1 = “strongly agree,” 4 = “strongly disagree”). Total scores range from 10 to 40, with higher scores reflecting higher self-esteem. The original RSES demonstrates excellent psychometric properties, including strong internal consistency and test–retest reliability (Rosenberg, 1989). The Chinese version has been widely used and validated in local populations. In the present study, this scale demonstrated sound reliability and validity (Cronbach’s *α* = 0.82; KMO = 0.86, cumulative variance explained 70.19%).

#### 3.3.3 Social anxiety scale

Social anxiety was evaluated using the Social Anxiety Scale (SAS), this 6-item social anxiety subscale is derived from the Self-Consciousness Scale (Fenigstein et al., 1975; Chinese version: Wang et al., 1999). In the original study, the social anxiety subscale showed good test–retest reliability over a two-week period (*r* = 0.73). Its validity as a distinct construct was supported by factor analysis, where it loaded separately from public and private self-consciousness, and it demonstrated expected correlations with measures of social discomfort (Fenigstein et al., 1975). This 6-item instrument assesses anxiety in social situations. Two typical items from the subscale are: “I feel nervous when I speak in front of a group” and “I find it easy to talk to strangers” (reverse-scored). Items are scored on a 5-point scale (0 = “extremely inconsistent,” 4 = “extremely consistent”), yielding a total score ranging from 0 to 24. Higher scores indicate greater social anxiety. Following validation, this scale demonstrated sound reliability and validity in the present study (Cronbach’s *α* = 0.82; KMO = 0.86, cumulative variance explained 57.09%).

#### 3.3.4 Exercise addiction scale

The risk of exercise addiction was assessed using the Exercise Addiction Inventory (EAI) (Griffiths et al., 2005; Chinese version: Zhang and Li, 2010). This 6-item scale evaluates six domains: salience, conflict, emotional adjustment, tolerance, withdrawal symptoms, and relapse. Responses are recorded on a 5-point Likert scale (1 = “strongly disagree,” 5 = “strongly agree”). The total score ranges from 6 to 30, with higher total scores indicating higher addiction risk. A typical item for each of two domains is: “Exercise is the most important thing in my life” (salience) and “I use exercise as a way of changing my mood” (mood modification). In the original validation study, the EAI demonstrated very good internal consistency (Cronbach’s *α* = 0.84). Its construct validity was supported by a principal components analysis confirming a single factor structure, and it successfully differentiated between individuals with high and low exercise frequency. Concurrent validity was established through strong correlations with other established measures, including the Obligatory Exercise Questionnaire (*r* = 0.80) and the Exercise Dependence Scale (*r* = −0.81) (Griffiths et al., 2005). Following validation, the scale demonstrated good reliability and validity in this study (Cronbach’s *α* = 0.81; KMO = 0.79, cumulative explained variance 71.33%).

### 3.4 Data analysis

The data analysis procedure comprised three steps:

1) Common method bias assessment: Harman’s single-factor test was conducted to assess potential common method bias. The first unrotated factor in exploratory factor analysis explained 32.10% of the variance, below the 40% threshold, indicating no significant common method bias.
2) Prior to the main analysis, key statistical assumptions were tested. The assessment of multivariate normality in Amos 24.0 yielded a Mardia’s coefficient of 30.51 with a critical ratio of 51.50 (*p* < 0.001), indicating a significant departure from normality. However, the primary mediation analysis utilized a bootstrap approach with 5,000 resamples, which is robust to such violations and ensures the reliability of the parameter estimates. Linearity and homoscedasticity were examined by visually inspecting scatterplots of standardized residuals against standardized predicted values for all key paths in the proposed model. These plots showed residuals randomly and evenly dispersed around zero, with no apparent systematic patterns, thus supporting the assumptions of linearity and homoscedasticity.
3) Descriptive statistics and correlations: Medians, Interquartile Range, and Spearman’s rank-order correlation coefficients were calculated for all variables (perfectionism, self-esteem, social anxiety, and exercise addiction).
4) Chained mediation analysis: Using the PROCESS macro (version 4.0) in SPSS 25.0, a chained mediation model was tested to examine the sequential pathways from perfectionism to exercise addiction via self-esteem and social anxiety. Gender, grade, and BMI were included as covariates. Bootstrap resampling (5,000 iterations) with 99% confidence intervals was applied to validate indirect effects.

## 4 Results

### 4.1 Common method bias test

As the data were collected via self-report questionnaires, we examined the threat of common method bias using Harman’s single-factor test. All items from the perfectionism, self-esteem, social anxiety, and exercise addiction scales were subjected to an exploratory factor analysis. The principal component analysis revealed that the first unrotated factor explained 33.87% of the total variance, which is below the recommended cutoff of 40%. Thus, significant common method variance was unlikely to have contaminated the results.

### 4.2 Descriptive statistics and correlations

Descriptive statistics and Spearman’s rank-order correlations were computed for the four variables: perfectionism, self-esteem, social anxiety, and exercise addiction (Table 1). All variables demonstrated significant intercorrelations:

**Table 1:** Descriptive statistics and correlation analysis of variables.

Variable	Median	Interquartile range	Perfectionism	Self-esteem	Social anxiety	Exercise addiction
Perfectionism	79	18(69, 87)	1			
Self-esteem	25	6(22, 28)	−0.55**	1		
Social anxiety	18	7(15, 22)	0.51**	−0.44**	1	
Exercise addiction	16	6(14, 20)	0.49**	−0.45**	0.42**	1

**p < 0.01.

Perfectionism was positively correlated with exercise addiction (*r* = 0.49, *p* < 0.01); Perfectionism showed a negative correlation with self-esteem (*r* = −0.55, *p* < 0.01) and a positive correlation with social anxiety (*r* = 0.51, *p* < 0.01); Self-esteem was negatively correlated with both exercise addiction (*r* = −0.45, *p* < 0.01) and social anxiety (*r* = −0.44, *p* < 0.01); Social anxiety was positively associated with exercise addiction (*r* = 0.42, *p* < 0.01).

### 4.3 Perfectionism and exercise addiction among university students: chain-mediated effects

To begin with, we assessed the overall fit of the proposed chained mediation model using Amos 24.0. The model was tested using maximum likelihood estimation. The results indicated a good fit to the data: *χ*^2^/df = 3.52, RMSEA = 0.037 (90% CI: 0.023–0.053), NFI = 0.997, RFI = 0.989, IFI = 0.998, TLI = 0.992, and CFI = 0.998. All fit indices met or exceeded established standards, suggesting that the model is acceptable for further analysis of the structural paths.

To further examine the predictive effects of perfectionism, self-esteem, and social anxiety on exercise addiction, hierarchical regression analyses were conducted. Perfectionism, self-esteem, and social anxiety were entered as independent variables, with exercise addiction as the dependent variable (Table 2). The results revealed significant effects:

**Table 2:** Regression analysis of perfectionism, self-esteem, and social anxiety on exercise addiction.

Variant	Physical activity			
	*β*	*T*	*F*	*R^2^*
Perfectionism	0.56	28.82***	830.50	0.31
Self-esteem	−0.45	−23.43***	548.77	0.23
Social anxiety	0.49	24.04***	578.08	0.24

***p < 0.001.

Perfectionism positively predicted exercise addiction (*β* = 0.56, t = 28.82, *p* < 0.001); Self-esteem negatively predicted exercise addiction (*β* = −0.45, *t* = −23.43, *p* < 0.001); Social anxiety positively predicted exercise addiction (*β* = 0.49, *t* = 24.04, *p* < 0.001).

Using perfectionism (X) as the independent variable, exercise addiction (Y) as the dependent variable, and self-esteem (W1) and social anxiety (W2) as the mediator variables, while controlling for demographic variables (gender, grade), we tested the chain mediation effect of self-esteem and social anxiety on the relationship between perfectionism and exercise addiction. The analysis was conducted using the PROCESS macro (version 3.4) in SPSS 25.0 to examine whether perfectionism exerts significant mediating and chain-mediated effects on exercise addiction. Model 6 in the PROCESS macro was employed to test the hypotheses, with 5,000 bootstrap samples and 95% confidence intervals (CIs) to validate the chain mediation effects.

From Table 3, perfectionism exhibited a significant positive effect on college students’ exercise addiction (*β* = 0.56, *p* < 0.001; 95% CI [0.129, 0.155]). This indicates that higher perfectionism scores predicted higher exercise addiction severity, confirming Hypothesis 1. Social anxiety mediated the relationship between self-esteem and exercise addiction (*p* < 0.001; 95% CI [0.014, 0.034]), suggesting that lower self-esteem increases exercise addiction through elevated social anxiety, thereby supporting Hypothesis 2. Self-esteem mediated the effect of perfectionism on exercise addiction (*p* < 0.001; 95% CI [0.015, 0.041]), indicating that perfectionism exacerbates exercise addiction by reducing self-esteem, which validated Hypothesis 3. Finally, self-esteem and social anxiety chain mediated the relationship (*p* < 0.001; 95% CI [0.003, 0.011]), demonstrating that perfectionism enhances exercise addiction through the chained pathway of diminished self-esteem and heightened social anxiety, thus confirming Hypothesis 4.

**Table 3:** Intermediary effect values and effect sizes.

Effect Path	Effect	Boot SE	Boot LLCI	Boot ULCI	Relative mediation effect
Total effect	0.14***	0.005	0.129	0.155	100%
Direct effect	0.08***	0.006	0.067	0.099	57.14%
Total indirect effect	0.06***	0.006	0.042	0.075	42.86%
Indirect effect 1 (self-esteem)	0.03***	0.005	0.015	0.041	21.43%
Indirect effect 2 (social anxiety)	0.02***	0.004	0.014	0.034	14.29%
Indirect effect 3 (self-esteem and social anxiety)	0.01***	0.002	0.003	0.011	7.14%

Indirect effect 1: perfectionism → self-esteem → exercise addiction; indirect effect 2: perfectionism → social anxiety → exercise addiction; indirect effect 3: perfectionism → self-esteem → social anxiety → exercise addiction. The symbol *** indicates statistical significance at *p* < 0.001.

**Figure 2:**
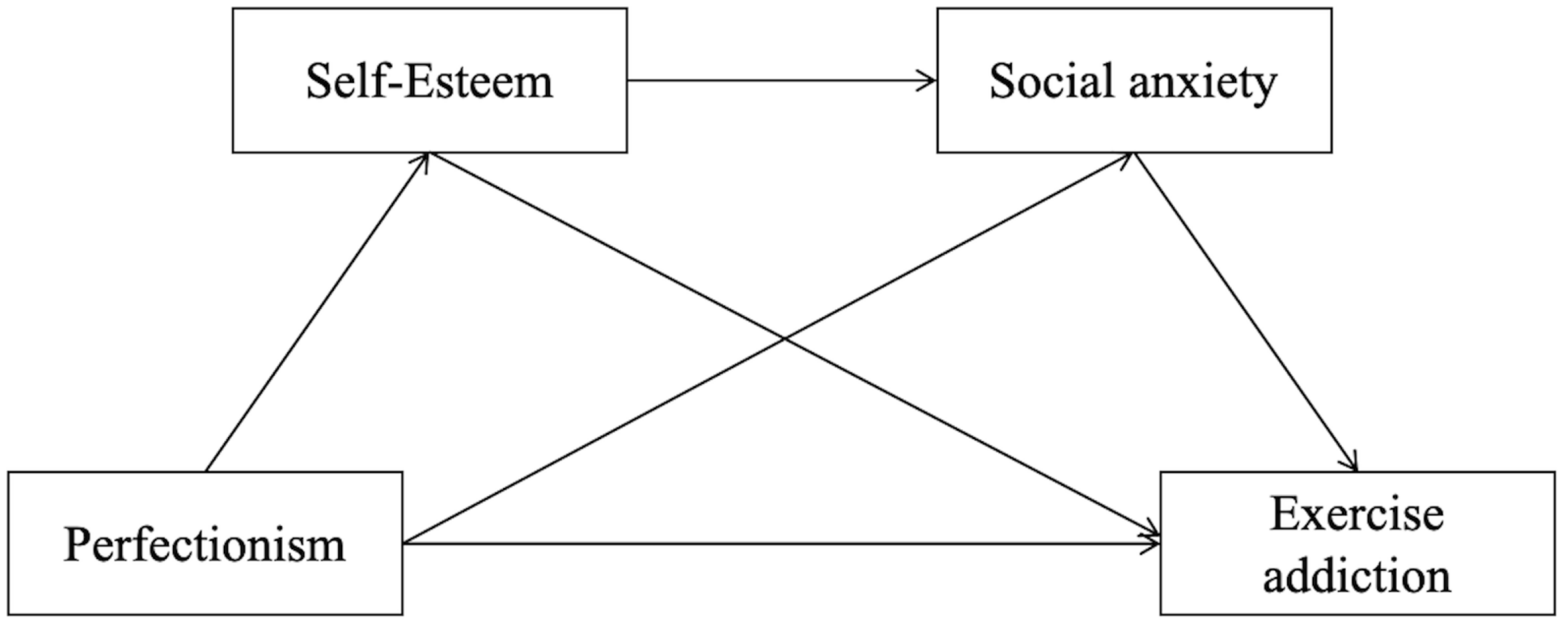
Model of mediating roles of self-esteem between perfectionism and exercise addiction.

**Figure 3:**
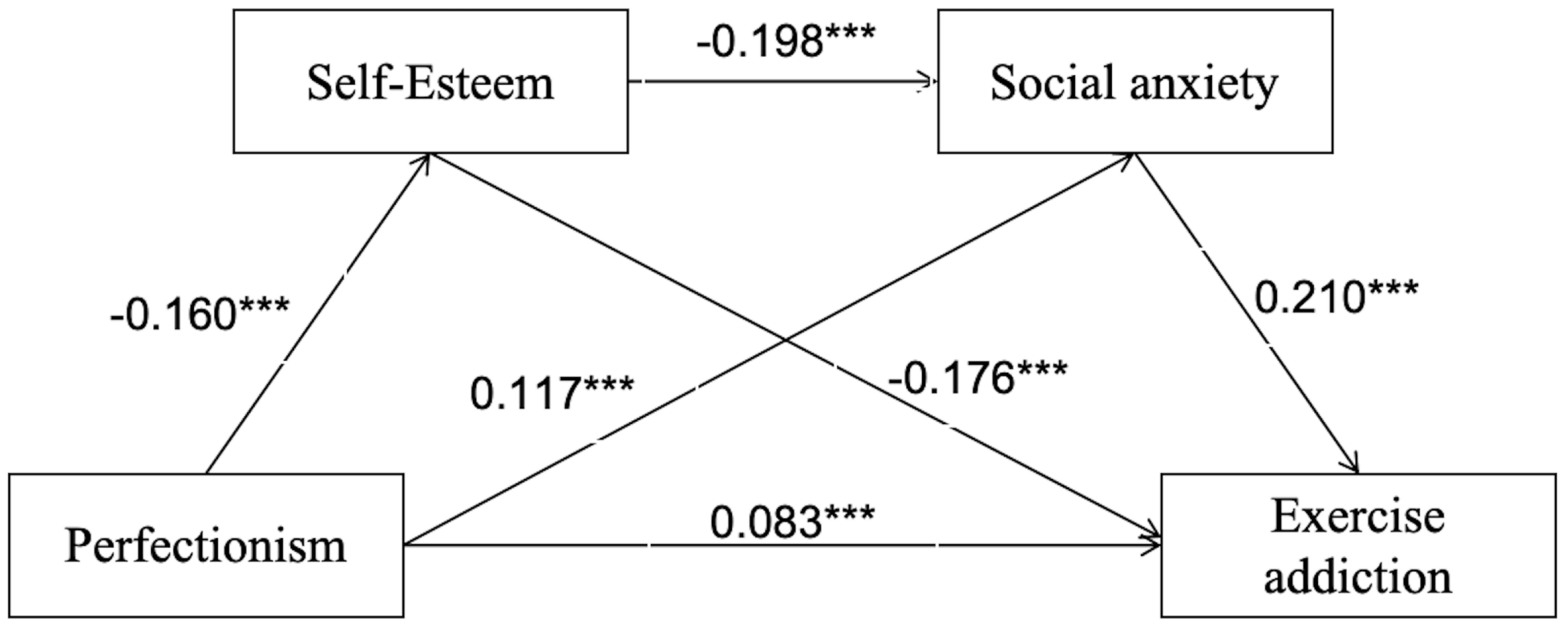
Model of mediating roles of social anxiety between perfectionism and exercise addiction. The symbol *** indicates statistical significance at *p* < 0.001.

**Figure 4:**
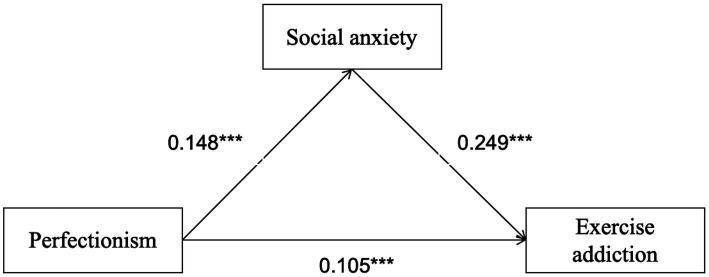
Model of chain mediating roles of self-esteem and social anxiety between perfectionism and exercise addiction. The symbol *** indicates statistical significance at *p* < 0.001.

Additionally, the effect sizes of the three mediation paths and the total effect were statistically significant (*p* < 0.001). The total effect value was 0.14, with a direct effect of 0.08 (57.14% of the total effect) and a total indirect effect of 0.06 (42.86%). Among the mediation pathways: Path 1 (perfectionism → self-esteem → exercise addiction), Effect = 0.03 (21.43% of total indirect effect); Path 2 (perfectionism → social anxiety → exercise addiction), Effect = 0.02 (14.29% of total indirect effect); Path 3 (perfectionism → self-esteem → social anxiety → exercise addiction), Effect = 0.01 (7.14% of total indirect effect). The specific paths are presented in Figures 2–4.

## 5 Discussion

The present study examined a theoretical model proposing that self-esteem and social anxiety chain mediate the relationship between perfectionism and exercise addiction among Chinese college students. The findings not only were consistent with the direct predictive effect of perfectionism on exercise addiction risk (H1) but also supported the potential roles of low self-esteem (H2), high social anxiety (H3), and their sequential transmission (H4) through a multiple mediation model. These results provide critical insights for the potential early identification and targeted intervention of exercise addiction, while offering preliminary cross-cultural evidence for mediating mechanisms in behavioral addiction research. However, the cross-sectional design necessitates caution in interpreting these associations as causal pathways.

### 5.1 The direct and indirect roles of perfectionism

The results demonstrated a significant direct association between perfectionism and exercise addiction (H1). This finding aligns with prior systematic reviews indicating that perfectionists often view exercise as a central strategy for maintaining self-worth and a sense of control (Çakın et al., 2021). Notably, the direct effect magnitude in this study was smaller than the total indirect effect of the mediated pathways, which is consistent with the notion that perfectionism primarily drives exercise addiction through psychological mechanisms. This finding resonates with Szabo’s “perfectionism trap” theory: when individuals excessively bind exercise performance to self-identity (e.g., “Only consistent exercise proves my worth”), they may become trapped in a vicious cycle of “pursuing excellence” and “self-punishment,” persisting in training despite physical harm or social conflicts (Szabo et al., 2018).

### 5.2 The mediating role of self-esteem

The significant independent mediation by self-esteem (H2) aligns with theoretical and empirical links between perfectionism, diminished self-worth, and excessive exercise (Guidi et al., 2013; Bardone-Cone et al., 2007). Perfectionists often report lower self-esteem, likely stemming from a perceived gap between their performance and their stringent standards. This state of low self-esteem is associated with specific compensatory patterns. For instance, individuals may engage in excessive exercise to reshape “body capital” (e.g., muscle mass or body fat percentage) to regain a sense of self-efficacy, or they may use exercise-induced endorphin release to temporarily buffer the distress of negative self-evaluations. Notably, literature suggests that unstable or contingent self-esteem—wherein self-worth is overly tied to exercise outcomes—may be particularly relevant in this pathway (Hall et al., 2009). When self-esteem is contingent on exercise, interruptions or declines in performance may lead to sharp drops in self-worth, which could in turn intensify compulsive exercise patterns—an important direction for future research.

### 5.3 The mediating role of social anxiety

Social anxiety also served as a significant independent mediator (H3), accounting for 14.29% of the indirect effect. This aligns with theoretical perspectives in which fear of negative social evaluation is associated with maladaptive behaviors. In the context of this study, perfectionism, potentially in conjunction with lower self-esteem, was correlated with heightened concerns about social judgment. From the perspective of self-regulation theory, the fear of negative social evaluation inherent in social anxiety not only causes distress but also consumes finite self-regulatory resources. This preoccupation with potential judgment can deplete the cognitive capacity needed for deliberate behavioral control. In this state of impaired self-regulation, exercise can become a default, yet ultimately maladaptive, strategy for managing anxiety and restoring a sense of control. This theoretical link helps explain the association between social anxiety and an increased risk of exercise addiction. This pattern may be particularly pronounced in cultural settings that emphasize a “presentable image,” such as China, where social evaluation and conformity represent salient values. Within such contexts, individuals are more likely to engage in excessive exercise to sculpt a physique perceived as socially desirable, thereby seeking acceptance and managing fears of exclusion. This interpretation corresponds with findings of a positive association between body image concerns and exercise addiction risk (Guo et al., 2025; Godoy-Izquierdo et al., 2023). It should be noted, however, that this coping pattern may be counterproductive. Although pursuing “body capital” might temporarily reduce feelings of social anxiety, the compulsive nature of over-exercise can itself contribute to social avoidance (e.g., skipping social events to train), potentially sustaining a cycle of anxiety, increased exercise, and further social impairment. This observed pattern echoes qualitative descriptions of exercise addiction as a “shield against social exclusion” that nevertheless accompanies long-term isolation (Lichtenstein et al., 2017) and is consistent with research linking exercise to coping with social anxiety and loneliness (Lukács et al., 2019), including in Chinese student samples (Li et al., 2015).

### 5.4 The chain mediation pathway

Statistical support was found for the chain mediation path (perfectionism → self-esteem → social anxiety → exercise addiction risk), explaining one multilevel psychological mechanism underlying exercise addiction risk in college students. The chained mediation effect accounted for 7.14% of the total effect, a small yet statistically significant portion. This situates the sequential pathway as one meaningful component within a broader network of psychological factors, underscoring the multifactorial nature of exercise addiction. Consequently, interventions targeting this specific pathway alone may have limited universal impact. However, its role could be more pronounced in high-risk subgroups, a possibility that should guide future hypothesis-driven research. This pattern can be interpreted through the lens of self-regulation theory. This pattern aligns with self-regulation theory: perfectionists’ self-worth depletion consumes cognitive resources, and when coupled with heightened social anxiety, impairs the conscious control needed to moderate exercise behavior. Within this framework, perfectionists’ “achievement orientation” leads to setting rigid and unrealistic exercise standards (Shafran et al., 2002). The perceived failure to meet these standards further depletes self-regulatory resources and erodes self-esteem. In collectivist cultural contexts that prioritize conformity and an “ideal body shape,” individuals with low self-esteem may overestimate the risk of negative social judgment. To manage the resulting anxiety and depleted self-control, they may increasingly rely on the predictable structure and immediate mood-altering effects of exercise. However, this reliance can escalate into compulsive use, leading to social withdrawal (Lichtenstein et al., 2017) and further regulatory breakdown, thus reinforcing a cycle of low self-esteem, anxiety, and excessive exercise. This aligns with the view that compulsive exercisers may trade long-term social and psychological well-being for short-term emotional relief and a sense of control (Szabo et al., 2018).

### 5.5 Limitations and future directions

The present study provided initial support for the chained mediation model of self-esteem and social anxiety, addressing the limitation of prior research that focused on single mediator variables. Despite its contributions, this study is subject to several limitations that warrant attention. First, the cross-sectional design prohibits definitive causal inferences. Although our model is grounded in established theory, longitudinal or experimental studies are necessary to corroborate the temporal precedence and causality of the proposed pathways (e.g., whether low self-esteem indeed predisposes individuals to social anxiety, which is in turn associated with higher levels of exercise addiction).

Second, the generalizability of our findings may be constrained by the specific sample characteristics. Our participants were Chinese university students, a population particularly vulnerable to body image concerns and academic pressure, which may amplify the studied relationships. The mechanisms identified might differ in clinical populations, older adults, or individuals from cultural contexts with different norms around exercise and perfectionism. Furthermore, our study did not account for a range of potentially influential demographic and confounding variables, such as socioeconomic status, marital status, social media usage, personality traits (e.g., neuroticism), or pre-existing mental health conditions (e.g., clinical anxiety or depression). These omitted variables could act as common causes or moderators, potentially confounding the observed relationships between perfectionism, mediators, and exercise addiction. For instance, socioeconomic status might influence access to fitness facilities and leisure time, thereby affecting exercise patterns independently of psychological factors. Additionally, this study did not account for common comorbidities of exercise addiction, such as eating disorders or major depression, which may share underlying vulnerabilities with the proposed model. Future research should investigate how these comorbid conditions interact with the perfectionism-self-esteem-social anxiety pathway.

Third, the reliance on self-report measures introduces the possibility of common method variance, although Harman’s single-factor test suggested it was not a critical issue in this dataset. Furthermore, self-reports are susceptible to other biases such as social desirability and recall accuracy, which could influence the responses.

These limitations chart a clear course for future research. Future studies should: Employ longitudinal designs to track the development of exercise addiction and establish causality over time. Replicate and extend this model in more diverse populations, including professional athletes (who may have different motivational profiles), members of private fitness clubs, older adults, and across different cultural settings to test its boundary conditions. Integrate a broader array of measures, including objective behavioral data (e.g., exercise logs from wearable devices), interviews for clinical assessment, and importantly, control for key confounding variables such as socioeconomic status, personality inventories, and diagnostic screening for mental disorders to isolate the unique effects of the proposed psychological mechanisms. Explore the role of modern contextual factors like social media use, which may exacerbate social comparisons and perfectionistic tendencies.

### 5.6 Practical implications

Given the cross-sectional and correlational nature of this study, the findings cannot support direct practical implementation. However, they offer preliminary insights to inform the design of future longitudinal and intervention research. Potential avenues include investigating universal prevention strategies for the student population, such as psychoeducation to promote balanced exercise attitudes and multidimensional self-worth, or environmental initiatives to foster body-positive campus cultures. For high-risk subgroups, future trials could explore the utility of early screening for perfectionism, as well as test the efficacy of targeted protocols that integrate cognitive restructuring with social skills training, aiming to disrupt the psychological pathways identified herein. The ultimate value and feasibility of any such strategies must be rigorously established through prospective experimental studies.

## 6 Conclusion

Based on cross-sectional data, this study examined the associations between perfectionism, self-esteem, social anxiety, and exercise addiction risk among Chinese university students. The results indicated a significant positive association between perfectionism and exercise addiction risk. Both self-esteem and social anxiety were found to independently mediate this relationship. A chain mediation pathway was also identified, suggesting that perfectionism may relate to increased exercise addiction risk through lower self-esteem and, in turn, higher social anxiety. Theoretically, this pattern aligns with the concept of limited self-regulatory resources, where social anxiety may deplete psychological resources, potentially compromising an individual’s ability to regulate exercise behavior. Overall, the findings highlight low self-esteem and high social anxiety as potential psychological pathways linking perfectionism to exercise addiction risk in this population. Collectively, these results provide support for the tested theoretical model but should be interpreted as model-testing rather than model-confirming, given the correlational nature of the data. Further longitudinal research is needed to clarify the directionality of these associations and to test the model in broader samples.

## Data Availability

The original contributions presented in the study are included in the article/Supplementary material, further inquiries can be directed to the corresponding author.
